# Ibrutinib synergizes with poly(ADP-ribose) glycohydrolase inhibitors to induce cell death in AML cells via a BTK-independent mechanism

**DOI:** 10.18632/oncotarget.6409

**Published:** 2015-11-27

**Authors:** Lianne E. Rotin, Marcela Gronda, Neil MacLean, Rose Hurren, XiaoMing Wang, Feng-Hsu Lin, Jeff Wrana, Alessandro Datti, Dwayne L. Barber, Mark D. Minden, Malik Slassi, Aaron D. Schimmer

**Affiliations:** ^1^ Princess Margaret Cancer Centre, Ontario Cancer Institute, University Health Network, Toronto, Ontario, Canada; ^2^ Institute of Medical Science, University of Toronto, Toronto, Ontario, Canada; ^3^ Samuel Lunenfeld Research Institute, Toronto, Ontario, Canada; ^4^ Department of Agricultural, Food, and Environmental Sciences, University of Perugia, Perugia, Italy; ^5^ Fluorinov Pharma Inc., Toronto, Ontario, Canada

**Keywords:** AML, BTK, ibrutinib, ethacridine lactate, PARG

## Abstract

Targeting Bruton's tyrosine kinase (BTK) with the small molecule BTK inhibitor ibrutinib has significantly improved patient outcomes in several B-cell malignancies, with minimal toxicity. Given the reported expression and constitutive activation of BTK in acute myeloid leukemia (AML) cells, there has been recent interest in investigating the anti-AML activity of ibrutinib. We noted that ibrutinib had limited single-agent toxicity in a panel of AML cell lines and primary AML samples, and therefore sought to identify ibrutinib-sensitizing drugs. Using a high-throughput combination chemical screen, we identified that the poly(ADP-ribose) glycohydrolase (PARG) inhibitor ethacridine lactate synergized with ibrutinib in TEX and OCI-AML2 leukemia cell lines. The combination of ibrutinib and ethacridine induced a synergistic increase in reactive oxygen species that was functionally important to explain the observed cell death. Interestingly, synergistic cytotoxicity of ibrutinib and ethacridine was independent of the inhibitory effect of ibrutinib against BTK, as knockdown of BTK did not sensitize TEX and OCI-AML2 cells to ethacridine treatment. Thus, our findings indicate that ibrutinib may have a BTK-independent role in AML and that PARG inhibitors may have utility as part of a combination therapy for this disease.

## INTRODUCTION

Ibrutinib is a small-molecule Bruton's tyrosine kinase (BTK) inhibitor approved for clinical use in several B-cell malignancies, including chronic lymphocytic leukemia (CLL). Inhibition of BTK induces cell death by blocking constitutive B-cell receptor (BCR) signaling and impairing tumor-microenvironment interactions in CLL cells [1, 2]. BTK is expressed in almost all B-hematopoietic malignancies, but is also expressed in myeloid cells and myeloid malignancies where it can be activated through mechanisms distinct from BCR signaling. Since BTK is expressed in myeloid cells, we evaluated ibrutinib in acute myeloid leukemia (AML).

AML is a hematologic malignancy characterized by the overproduction of poorly differentiated myeloid-lineage cells [3]. Previously, other groups reported increased expression and constitutive activation of BTK in AML cell lines and primary AML patient samples [4–8]. BTK mediates signal transduction from the FLT3-ITD, TLR9 and CXCR4 receptors in AML cell lines, thereby promoting leukemic cell survival, growth, and migration [7, 9]. We further characterized the anti-AML activity of ibrutinib and identified drugs that sensitize AML cells to ibrutinib. Through exploration of the synergistic activity of ibrutinib with other drugs, we uncovered a BTK-independent role for ibrutinib with potential clinical utility in AML.

## RESULTS

### BTK is overexpressed and constitutively active in AML cells

**Figure 1:**
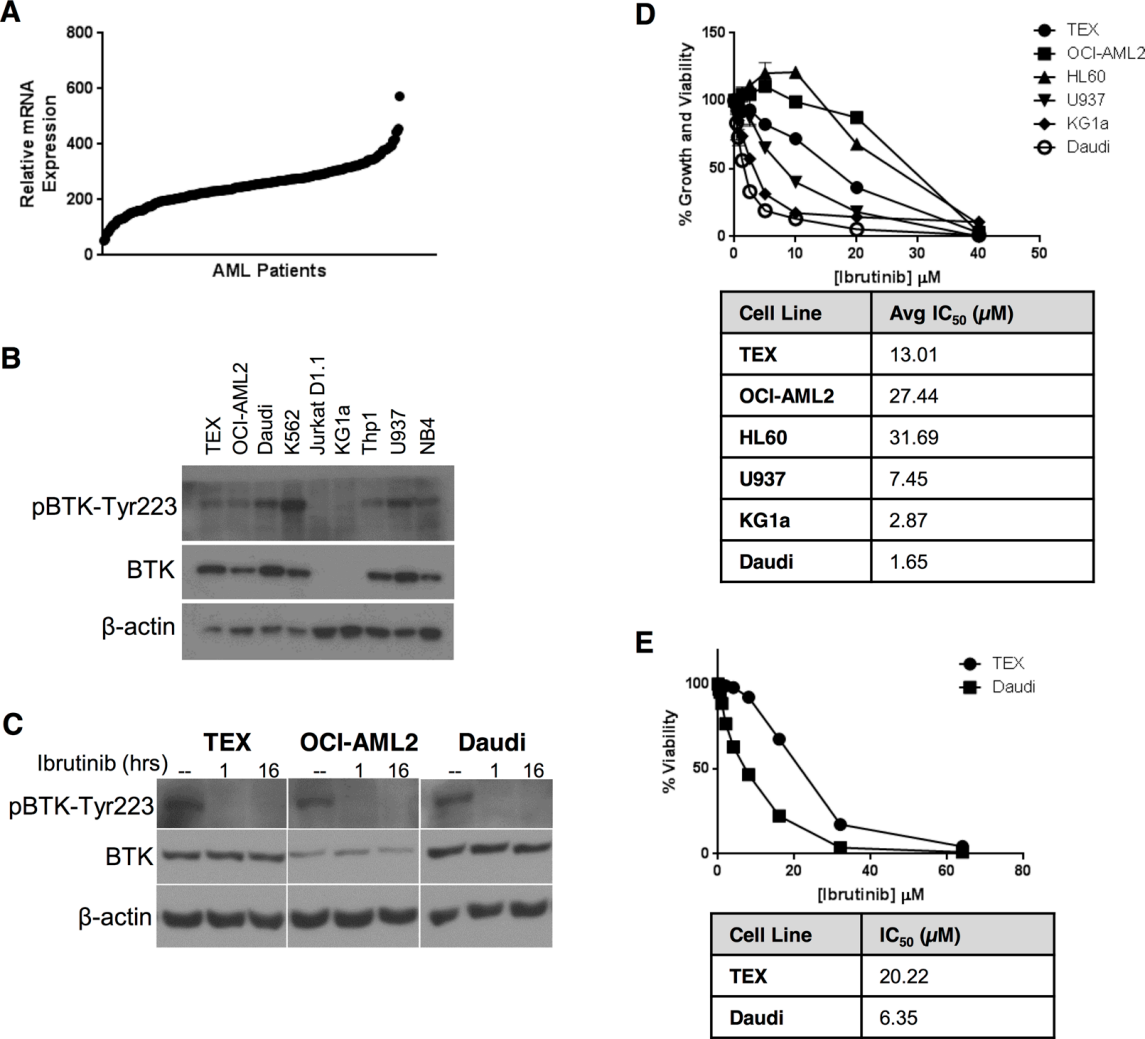
AML cell lines express constitutively active BTK, but are insensitive to ibrutinib (**A**) BTK mRNA expression in primary AML cells was determined by analysis of microarray gene expression (GEO accession code: GSE1159). BTK mRNA expression was examined in 267 patients with AML [46]. (**B**) BTK and pBTK-Y223 expression in AML cell lines was determined by immunoblotting. (**C**) TEX and OCI-AML2 cells were treated with 1 μM ibrutinib for 1 h or 16 h. pBTK-Y223 and BTK expression in cell lysates was detected by immunoblotting. (**D**, **E**) AML cell lines were treated with increasing concentrations of ibrutinib over 72 h and cell growth and viability relative to untreated cells was determined by (D) Alamar Blue or (E) Annexin V and PI staining on flow cytometry. Data depict mean relative viability ± SD from a representative experiment performed in triplicate. Data are representative of three (D) or two (E) independent experiments.

To determine the relevance of BTK as a therapeutic target in AML, we examined the protein and mRNA expression of BTK in a panel of AML cell lines. Analysis of the Cancer Cell Line Encyclopedia [4] demonstrated that AML cells expressed levels of BTK mRNA similar to B-cell malignancies (Figure S1). Likewise, a subgroup of primary AML patient samples had increased BTK expression (Figure 1A). Next, we evaluated the expression of BTK by immunoblotting in a series of AML cell lines. OCI-AML2, THP1, U937, NB4, K562, and the stem cell-like AML cell line TEX all expressed BTK, but this protein was not detectable in KG1a AML cells or Jurkat D1.1 T-ALL cells (Figure 1B). Phosphorylation of BTK at Tyr223, a marker of BTK activation [10–12], was detected in all cell lines expressing BTK (Figure 1B), suggesting constitutive BTK activity.

### AML cell lines are insensitive to chemical BTK inhibition with ibrutinib

To investigate the effects of BTK inhibition on AML viability and proliferation, we treated AML cells with increasing concentrations of ibrutinib. Phospho-BTK was reduced to undetectable levels at doses as low as 1 μM ibrutinib (Figure 1C). Compared to the sensitive B-lymphoblastic leukemia cell line, Daudi, the AML cell lines TEX, OCI-AML2, HL60, and U937 were relatively insensitive to ibrutinib, with IC_50_s ranging from 4- to 30-fold higher than Daudi cells in the Alamar Blue Assay, which measures cell proliferation and viability (Figure 1D) and much higher than the 1 μM concentration required to reduce levels of phospho-BTK. Similar insensitivity to ibrutinib was seen when measuring cell viability with Annexin V and PI staining (Figure 1E), which measures apoptosis. Interestingly, KG1a cells lacking detectable expression of BTK were the most sensitive to ibrutinib compared to other AML cell lines (KG1a IC_50_ = 2.87 μM by Alamar Blue assay).

### A combination chemical screen with ibrutinib in AML cell lines identifies the PARG inhibitor, ethacridine lactate, as an ibrutinib sensitizer

**Figure 2:**
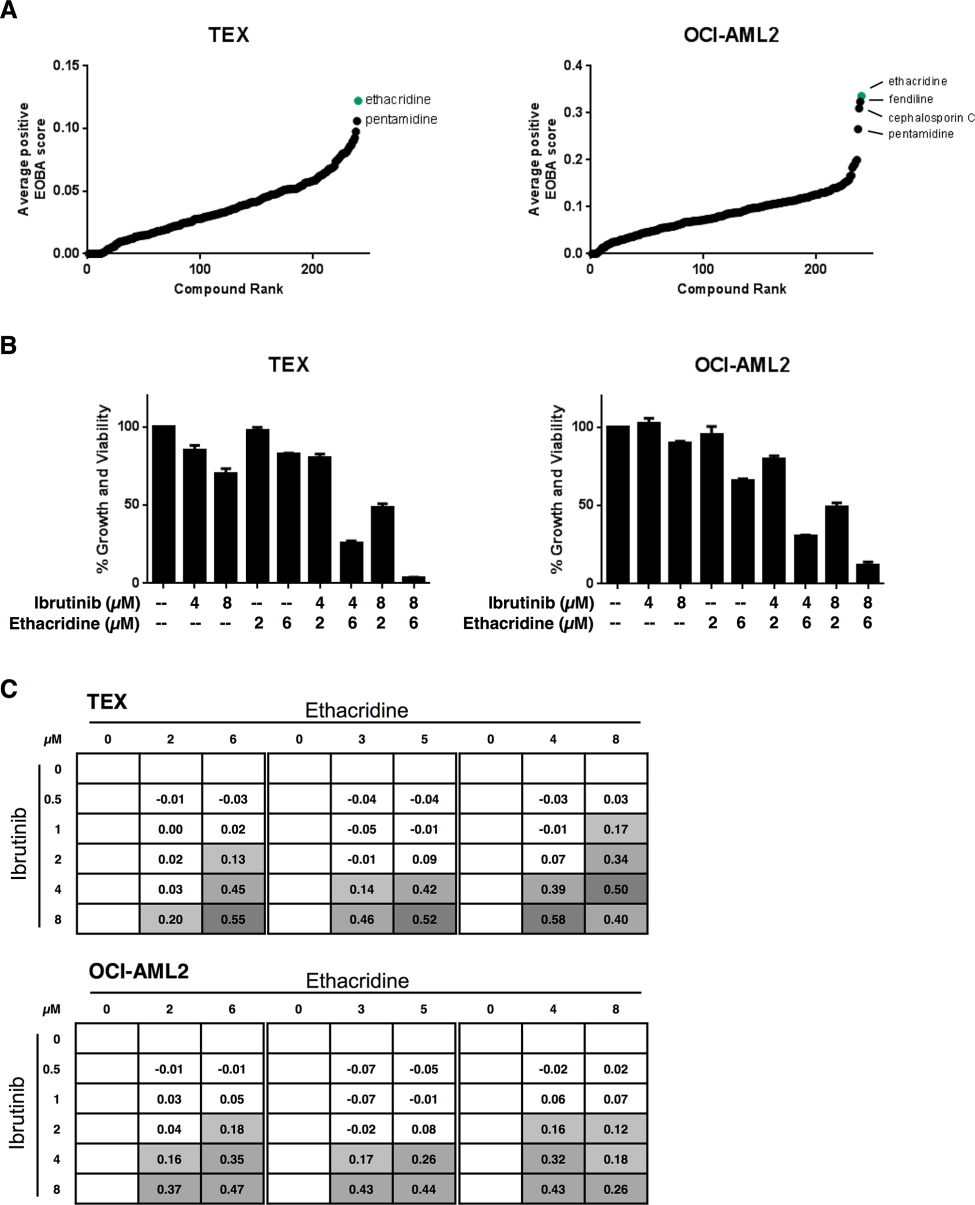
The PARG inhibitor ethacridine lactate sensitizes AML cell lines to ibrutinib (**A**) Ibrutinib was co-treated with 240 drugs in TEX and OCI-AML2 cells for 72 h. Growth and viability was determined with the SRB assay and synergy was calculated using the EOBA formula as described in the methods. Compounds were ranked in order of increasing average positive EOBA score. (**B**, **C**) TEX and OCI-AML2 cells were combination-treated with ibrutinib and ethacridine for 72 h and cell growth and viability relative to untreated cells was determined by Alamar Blue. (B) Data represent mean growth and viability ± SD from a representative experiment performed in triplicate. (C) EOBA synergy scores (shown) were calculated for each of the combinations tested. EOBA values > 0.1 (lightest grey) denote a synergistic combination, while values > 0.5 (darkest grey) denote a strongly synergistic combination. Data represent mean EOBA scores from a representative experiment performed in triplicate.

Given the limited single-agent cytotoxicity in AML cell lines, we sought to determine whether we could identify drugs that sensitize AML cells to ibrutinib. To this end, we carried out a high-throughput combination chemical screen with ibrutinib in both TEX and OCI-AML2 cells. The cell lines were co-treated with ibrutinib and increasing concentrations of compounds from an in-house chemical library containing 240 internationally prescribed drugs for a total of 5046 different assays among the two cell lines in this screen. Following 72 hours of incubation, cell growth and viability were measured by the sulforhodamine-B (SRB) assay. Potential synergistic hits were identified using the excess-over-Bliss additivism (EOBA) formula and average positive EOBA scores for each combination were rank ordered (Figure 2A). Ethacridine lactate and pentamidine were top synergistic hits common to both TEX and OCI-AML2 cells. We validated both combinations in these cell lines, but pursued ethacridine lactate over pentamidine because of its greater synergy with ibrutinib (EOBA scores of up to 0.58 and 0.47 by Alamar Blue in TEX and OCI-AML2, respectively) (Figure 2B, Figure S2). The ibrutinib-ethacridine combination induced cell death, as determined by Annexin V and PI staining, but the mechanism of cell death was caspase-independent (Figure S3). Ibrutinib and ethacridine also induced strong synergistic cytotoxicity in U937, HL60, and K562 leukemia cells, but not in KG1a cells (Figure S4).

Ethacridine lactate is used clinically as a topical antiseptic [13] and intra-amniotic abortifacient [14]. It is a DNA intercalator and putative poly(ADP-ribose) glycohydrolase (PARG) inhibitor [15, 16].

### The ibrutinib-ethacridine combination is preferentially cytotoxic to a subset of primary AML cells compared to normal hematopoietic cells

**Figure 3:**
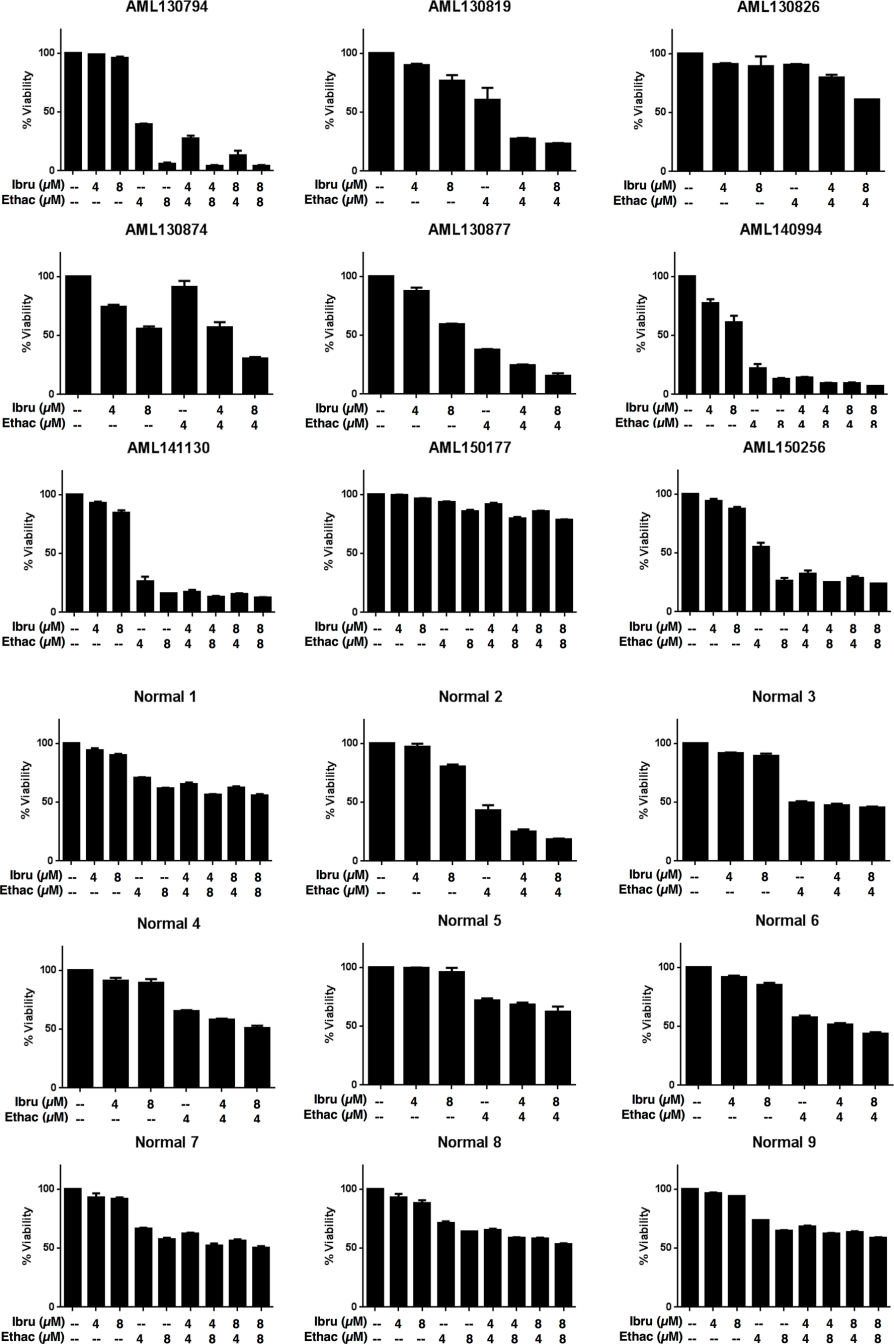
The ibrutinib-ethacridine combination is preferentially cytotoxic to primary AML cells over normal hematopoietic cells Primary AML and normal hematopoietic cells (G-CSF mobilized peripheral blood stem cells) were treated with ibrutinib, ethacridine, or both in combination for 48 h. Viability was determined by Annexin V and PI staining. Data represent mean percent viability ± SD from a single experiment performed in triplicate. Ibru = ibrutinib, Ethac = ethacridine.

Having identified the combination of ethacridine and ibrutinib as synergistic in AML cell lines, we tested the combination in primary AML cells (*n* = 9) (see Supplementary Table 1 for patient characteristics) and normal hematopoietic cells obtained from consenting donors of G-CSF mobilized stem cells for allotransplantation (*n* = 9). Primary cells were incubated with increasing concentrations of ethacridine and ibrutinib for 48 hours in Iscove's Modified Dulbecco's Medium supplemented with 10% fetal bovine serum, without additional growth factors, and viability was subsequently measured with Annexin V/PI staining and flow cytometry (Figure 3). Similar to the AML cell lines, ibrutinib had minimal single-agent cytotoxicity, with IC_50_s exceeding 8 μM in all primary cells. We noted that primary AML cells, on average, were more sensitive to single-agent ethacridine and combination ibrutinib-ethacridine treatment compared to normals: a subset of 6 of 9 AMLs demonstrated greater than 70% cell death from the combination, while only 1 of 9 normals (Normal 2) exhibited similar sensitivity. However, in some normal samples, the drug combination induced ≥ 50% cell death, suggesting that the ibrutinib-ethacridine combination may also have toxicity towards some normal hematopoietic cells.

### The combination of ibrutinib and ethacridine delays the growth of AML cells *in vivo*

**Figure 4:**
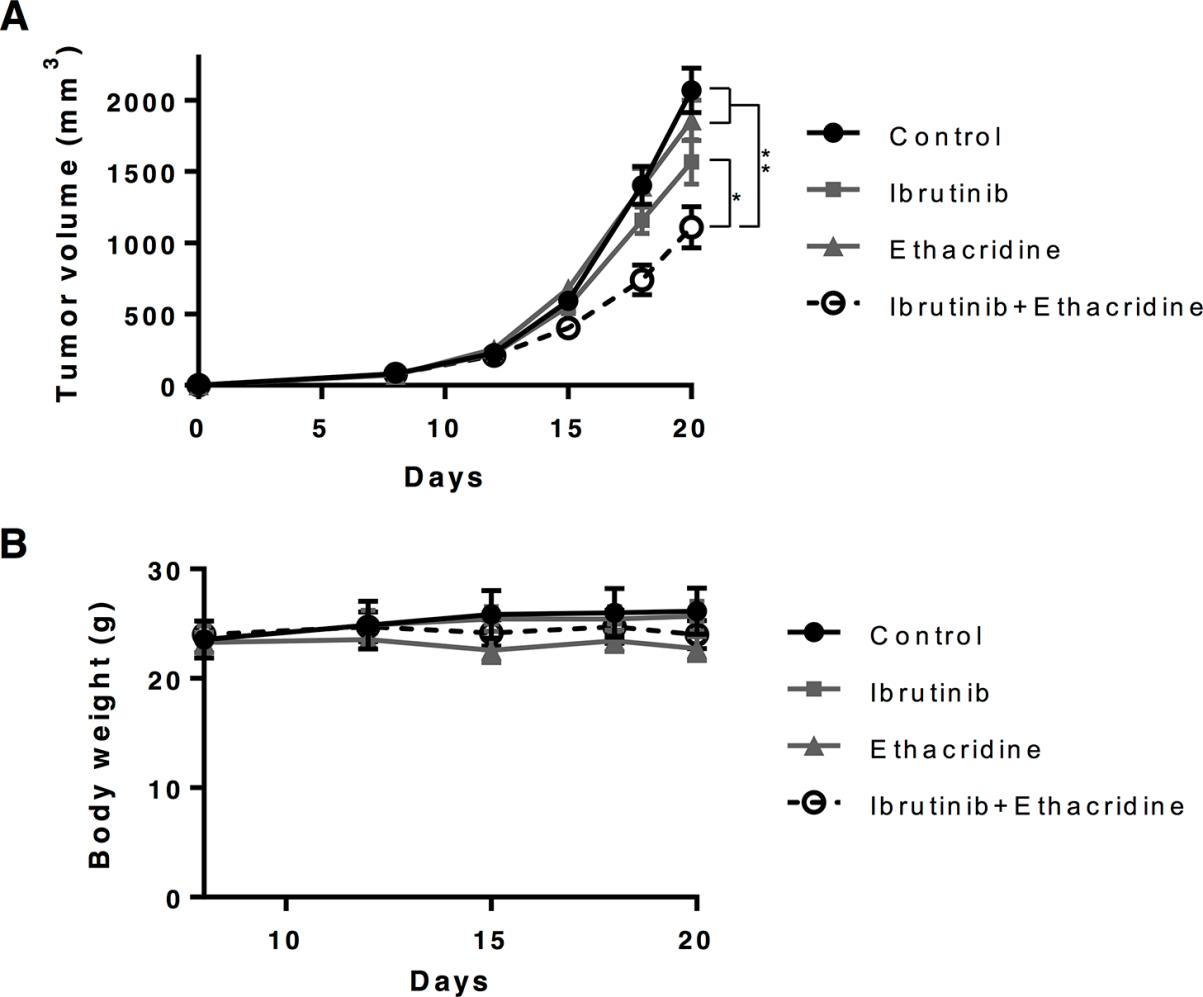
Ibrutinib-ethacridine combination displays anti-AML activity in mice 1 × 10^6^ OCI-AML2 cells were subcutaneously injected in SCID mice. Eight days after injection, mice were treated with 300 mg/kg of ibrutinib by oral gavage, 20 mg/kg of ethacridine by i.p. injection, a combination of two drugs, or vehicle control (5% DMSO, 20% Cremophor, 0.9% NaCl) by oral gavage on the indicated days. Tumor volume (**A**) and body weight (**B**) were monitored over time. Mean ± SEM for tumor volume and mean ± SD for body weight, *n* = 7. *P < 0.001 and **P < 0.0001 from a two-way ANOVA with Tukey's posttests comparing all treatment groups at day 18 and 20.

To assess the *in vivo* efficacy and toxicity of ibrutinib in combination with ethacridine, we evaluated this combination in a mouse model of leukemia. SCID mice were injected subcutaneously with OCI-AML2 cells. When tumors were palpable, mice were treated with ibrutinib, ethacridine, or the combination of both drugs. The combination of ibrutinib and ethacridine decreased the growth of OCI-AML2 cells more than either drug alone (*P < 0.001 and **P < 0.0001). Of note, no toxicity from combination treatment was detected as measured by changes in body weight, behavior or gross examination of the organs at the end of the experiment (Figure 4).

### Ethacridine synergizes with other small molecule BTK inhibitors, but not inhibitors of unrelated kinases

**Figure 5:**
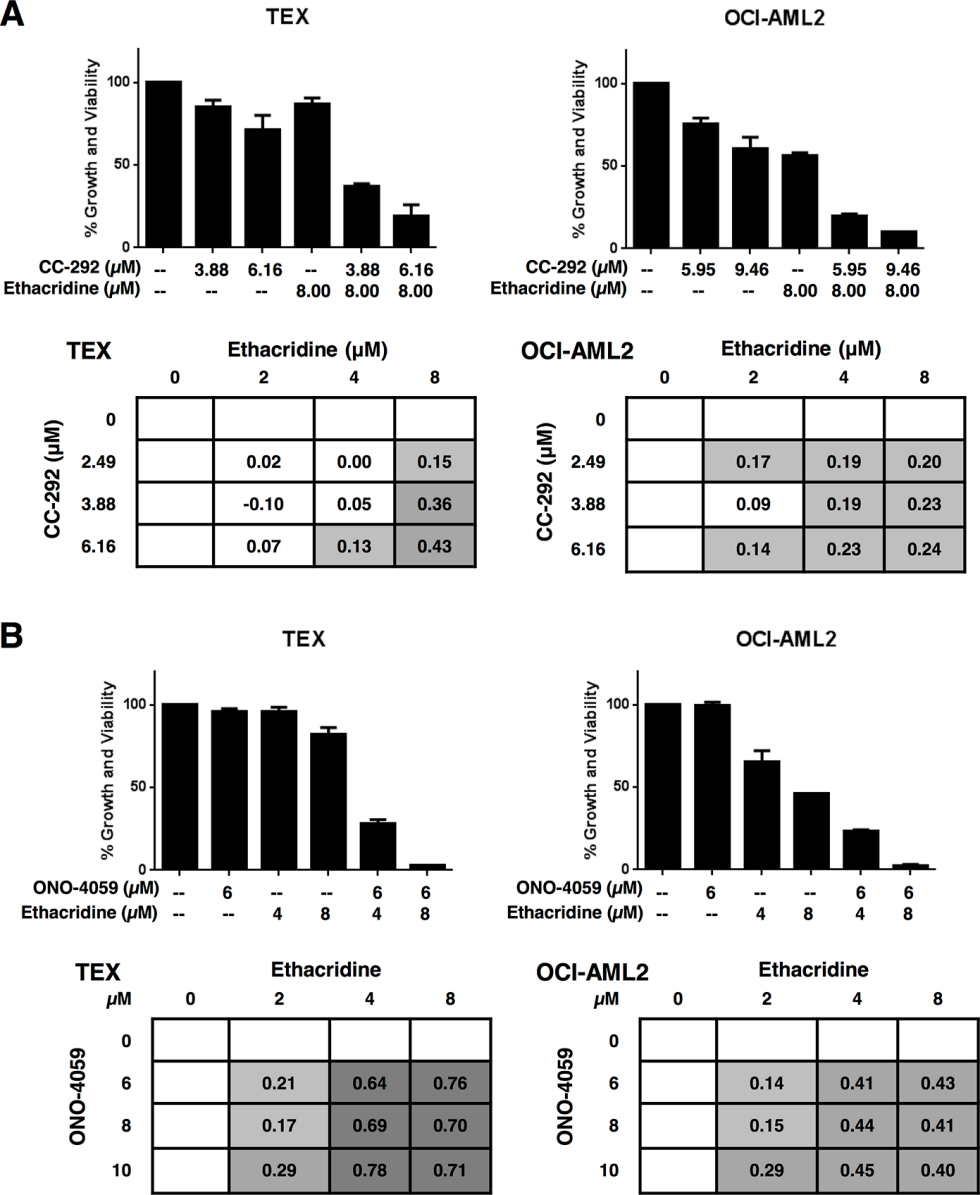
Ethacridine synergizes with other small-molecule BTK inhibitors TEX and OCI-AML2 cells were treated with increasing concentrations of ethacridine and (**A**) CC-292 or (**B**) ONO-4059 for 72 h. Growth and viability was measured by Alamar Blue and EOBA synergy scores were calculated. Data depict mean percent viability ± SD and mean EOBA scores from a representative experiment performed in triplicate. Data are representative of three independent experiments.

We sought to investigate whether the observed synergy with ethacridine was specific to ibrutinib or a property common to other BTK inhibitors. We therefore tested ethacridine in combination with two other BTK inhibitors currently in clinical trials: CC-292 and ONO-4059. Cell growth and viability was measured 72 hours after incubation by the Alamar Blue assay and EOBA scores were calculated. CC-292 and ONO-4059 synergized with ethacridine in TEX and OCI-AML2 cells with efficacy similar to ibrutinib (Figure 5).

**Figure 6:**
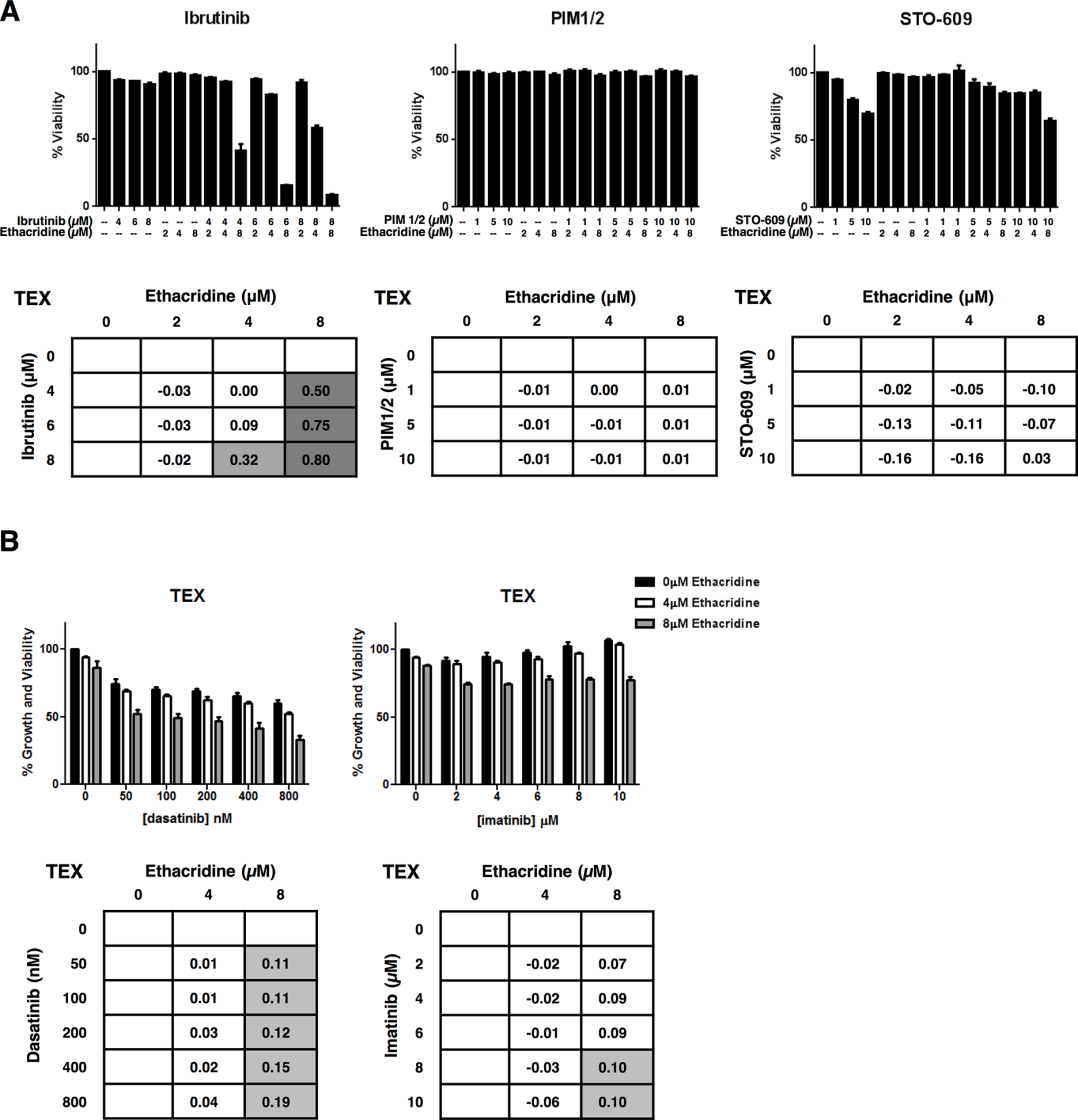
Ethacridine does not synergize with inhibitors of unrelated kinases (**A**) TEX cells were combination-treated with ethacridine and PIM1/2 or STO-609 for 72 h. Viability was measured by Annexin V/PI staining and EOBA scores were generated. Combination ibrutinib-ethacridine treatment of TEX cells was included as a positive synergy control for this method of cell viability determination. (**B**) TEX cells were combination-treated with ethacridine and dasatinib or imatinib for 72 h. Growth and viability was measured by Alamar Blue and EOBA synergy scores were calculated. Data depict mean percent viability (A) or growth and viability (B) ± SD and mean EOBA scores from a representative experiment performed in triplicate. Data are representative of three independent experiments.

To further examine the specificity of the synergistic activity of ethacridine, we sought to determine whether this compound generally sensitized AML cells to kinase inhibitors. We therefore selected inhibitors of kinase targets bearing minimal sequence similarity to BTK. Specifically, we tested PIM1/2 and STO-609, inhibitors of Calcium/calmodulin-dependent protein kinase family members PIM 1/2 and CaMKK, respectively. TEX cells were treated with these compounds in combination with ethacridine. Synergy was assessed by EOBA calculation following viability determination at 72 hours with Annexin V and PI staining on flow cytometry. Neither PIM1/2, nor STO-609 synergized with ethacridine in TEX cells, with EOBA scores not exceeding 0.03 for either combination (Figure 6A).

We also tested the combination of ethacridine with the ABL kinase inhibitor imatinib and the ABL and SRC family kinase inhibitor, dasatinib. Of note, ibrutinib is reported to inhibit SRC family kinases [17] as they share sequence homology to the TEC kinases. TEX and OCI-AML2 cells were combination-treated with ethacridine and these kinase inhibitors. Following a 72-hour incubation, cell growth and viability was determined by the Alamar Blue assay. The combinations produced primarily additive effects as calculated by the EOBA formula (Figure 6B and Figure S5). Thus, the observed synergy with ethacridine appears specific for TEC family kinase inhibitors.

### Ibrutinib and ethacridine synergize to induce cell death via a ROS-dependent mechanism

**Figure 7:**
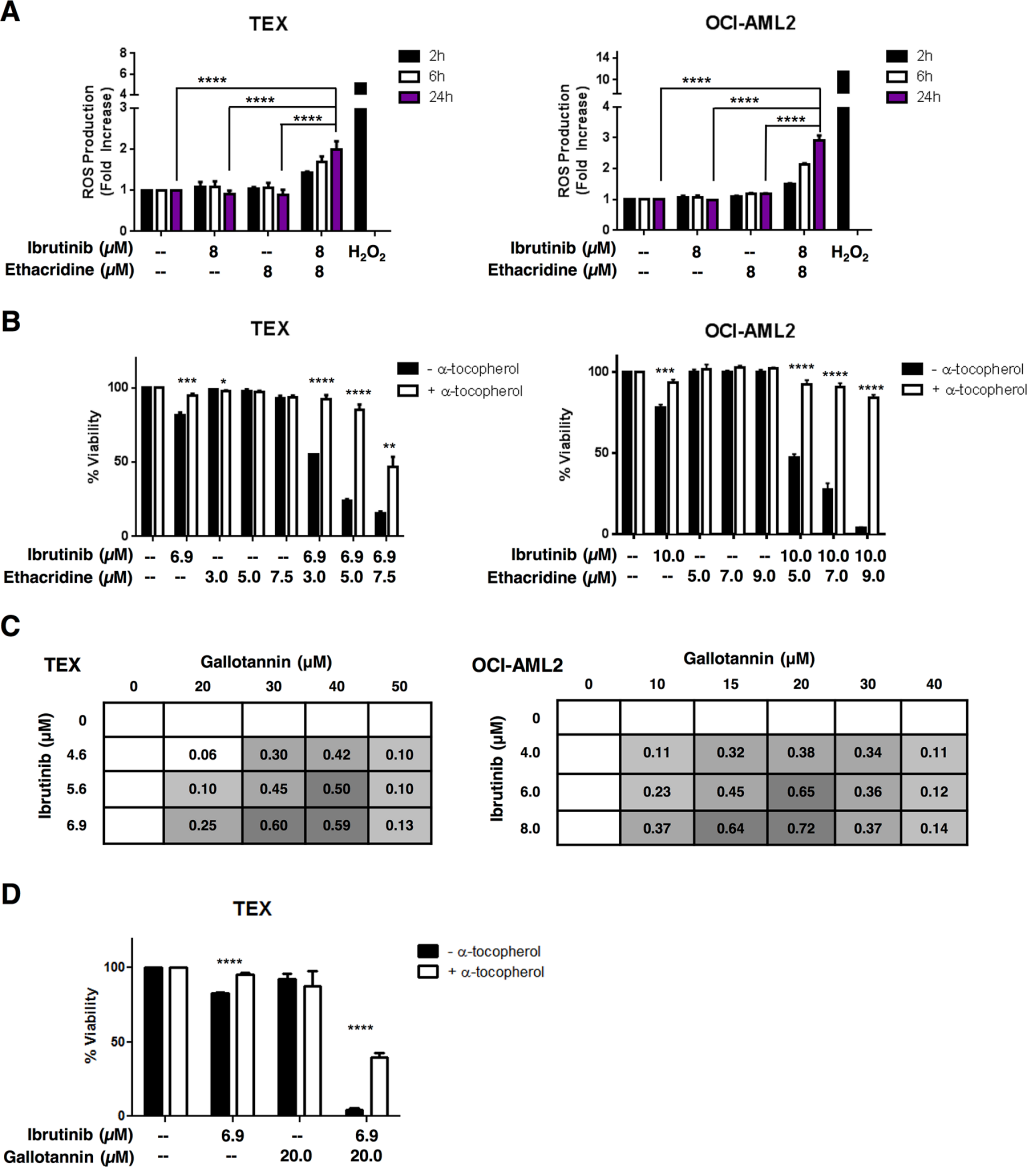
The ibrutinib-ethacridine combination induces cytotoxic levels of intracellular ROS (**A**) TEX and OCI-AML2 cells were treated with ibrutinib, ethacridine, or both in combination for 2, 6 or 24 h. Intracellular ROS was measured by carboxy-H_2_DCFDA staining and dead cells were excluded by PI staining on flow cytometry. Fold increase in ROS was calculated relative to the geometric means of carboxy-H_2_DCFDA (FITC) staining in untreated cells. H_2_O_2_ treatment served as a positive control for intracellular ROS generation. (**B**) TEX and OCI-AML2 cells were pre-treated with α-tocopherol prior to a 48 h incubation with ibrutinib and/or ethacridine. Viability was measured by Annexin V and PI staining, and calculated relative to respective untreated controls (+ or − α-tocopherol). (**C**) TEX and OCI-AML2 cells were treated with increasing concentrations of ibrutinib and gallotannin for 48 h. Viability was measured by Annexin V and PI staining and EOBA scores were calculated. Data represent mean EOBA scores from a representative experiment performed in triplicate. Data are representative of two (OCI-AML2) or three (TEX) independent experiments. (**D**) TEX cells were pre-treated with α-tocopherol and subjected to 48 h treatment with ibrutinib and gallotannin. Viability was measured by Annexin V and PI staining and calculated relative to untreated controls. Data represent mean fold increase in ROS production ± SD (A) or mean viability ± SD (B, D) from representative experiments performed in triplicate. Data are representative of two (A) or three (B, D) independent experiments. In all panels, *P < 0.05, **P < 0.01; ***P < 0.001; ****P < 0.0001 as determined by one-way ANOVA with Tukey's post-hoc test for multiple comparisons (A), or unpaired Student's *t* test with Holm-Sidak correction for multiple comparisons (alpha = 5%) (B, D).

To investigate the mechanism of synergy between ethacridine and ibrutinib, we examined ROS (reactive oxygen species) production in AML cells treated with the drug combination. Using carboxy-H_2_DCFDA staining and flow cytometry, we measured total intracellular ROS production after TEX and OCI-AML2 treatment with ibrutinib, ethacridine or the drug combination. At concentrations that were associated with synergistic cell death, neither drug alone markedly increased intracellular ROS production. However, ROS production in live cells was increased with the drug combination as early as two hours following treatment in both TEX and OCI-AML2 cells (Figure 7A). Moreover, the increased ROS production was functionally important for the observed cell death, as the addition of the anti-oxidant α-tocopherol abrogated cytotoxicity from the combination in both cell lines (Figure 7B). The observed increase in ROS following combination treatment did not appear to be mitochondrial in origin, as MitoSOX staining did not increase following combination treatment relative to single-agent treatment (Figure S6). Thus, these findings suggest that the synergistic cytotoxicity caused by the ibrutinib-ethacridine combination is due to excessive intracellular, but not mitochondrial, ROS production.

### The chemical PARG inhibitor gallotannin also synergizes with ibrutinib to induce cell death by excessive ROS production

Ethacridine is a putative PARG inhibitor [15, 16] and we demonstrated that ethacridine inhibited PARG (Figure S7). Therefore, we evaluated the combination of ibrutinib and gallotannin, another reported PARG inhibitor [18–20]. We treated TEX and OCI-AML2 cells with increasing concentrations of ibrutinib and gallotannin over 48 hours and then measured viability with Annexin V and PI staining. The ibrutinib-gallotannin combination was also profoundly synergistic, yielding EOBA values of up to 0.60 and 0.72 in TEX and OCI-AML2 cells, respectively (Figure 7C). Likewise, pre-treatment with α-tocopherol abrogated ibrutinib-gallotannin cytotoxicity (Figure 7D). Pretreatment with the poly(ADP-ribose) polymerase (PARP) inhibitor olaparib did not rescue combination-induced cytotoxicity (Figure S8). However, olaparib was directly toxic to the cells, thus potentially obscuring any protective effects.

### The synergy of ibrutinib with ethacridine is independent of the inhibitory effect on BTK

**Figure 8:**
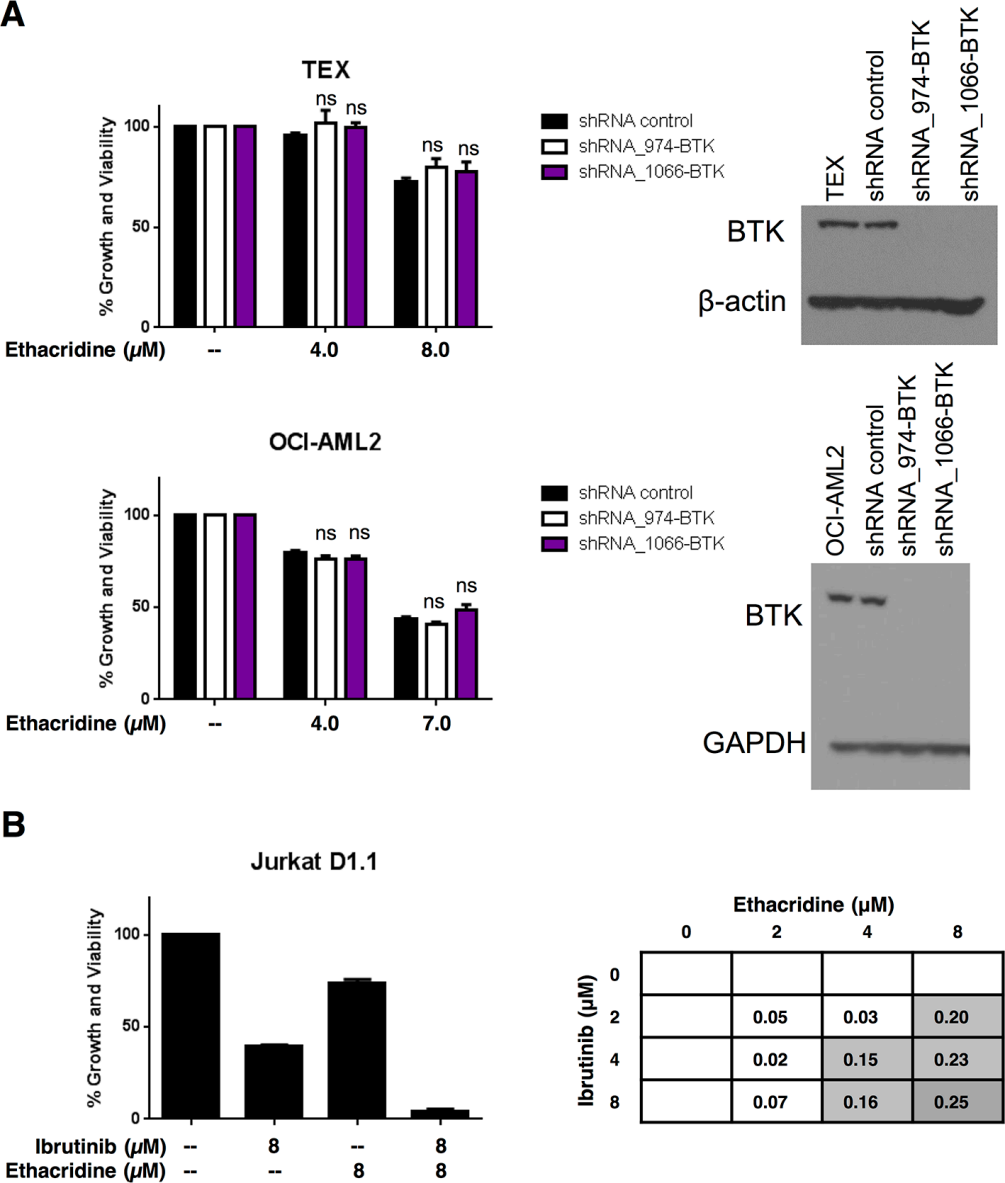
Ibrutinib's synergy with ethacridine is independent of BTK (**A**) TEX and OCI-AML2 cells were transduced with 2 different shRNAs targeting BTK or a non-targeting shRNA control in lentiviral vectors. On day 4 post-transduction, cells were treated with ethacridine at concentrations previously shown to synergize with ibrutinib. Growth and viability at 72 h post-ethacridine treatment was determined by Alamar Blue and calculated relative to untreated control. BTK knockdown was confirmed by immunoblotting. (**B**) Jurkat cells were treated with increasing concentrations of ibrutinib and ethacridine for 72 h. Cell growth and viability was determined by the Alamar Blue assay and synergy was calculated using the EOBA formula. Data depict mean percent growth and viability ± SD from a representative experiment performed in triplicate. Data in (A) and (B) are representative of two independent experiments. In all panels, ns = not significant, based on the results of an unpaired Student's *t* test with Holm-Sidak correction for multiple comparisons (alpha = 5%).

To determine whether synergy between ibrutinib and ethacridine was due to BTK inhibition by ibrutinib, we knocked down BTK with shRNA in TEX and OCI-AML2 cells. The reduction of BTK expression was confirmed by immunoblotting (Figure 8A) and qPCR (not shown). Knockdown cells were then treated with increasing concentrations of ethacridine and cell growth and viability was assessed by Alamar Blue. Despite substantial levels of BTK knockdown by shRNA, ethacridine treatment of BTK-knockdown cells was no more cytotoxic than ethacridine treatment of shRNA control cells (Figure 8A). These observations suggest that synergy of ibrutinib with ethacridine is independent of its inhibitory effect on BTK.

To further examine whether synergy of ibrutinib with ethacridine is due to targets beyond BTK, we tested the drug combination in Jurkat D1.1 cells, a T-acute lymphoblastic leukemia cell line that does not express BTK (Figure 1B). The ibrutinib-ethacridine combination synergized in Jurkat D1.1 cells, reaching EOBA values of 0.25 (Figure 8B), further supporting a synergistic mechanism for ibrutinib and ethacridine beyond AML cell lines analyzed in this study.

## DISCUSSION

The small molecule BTK inhibitor ibrutinib has demonstrated exceptional efficacy with minimal toxicity in several B-cell cancers. Ibrutinib is currently approved for clinical use in CLL, mantle-cell lymphoma (MCL) and Waldenström's macroglobulinemia (WM), owing to its impressive patient response rates during recent clinical trials. With widespread BTK expression across B-cell malignancies and its role as a node in several oncogenic signaling pathways, this cytoplasmic kinase has emerged as an attractive therapeutic target for B-lymphoid cancers. Multiple clinical trials investigating ibrutinib alone and in combination for these diseases are currently underway.

In addition to its expression in B-cell malignancies, BTK is also expressed in myeloid cell lines and can be activated through mechanisms independent of the B-cell receptor [7, 21, 22]. Thus, targeting BTK with ibrutinib may have efficacy in myeloid malignancies such as AML.

In concordance with previous work by Rushworth et al. [6] and Oellerich et al. [7], we demonstrated the expression of constitutively active BTK in several AML cell lines. However, in contrast to these other studies, in the cell lines we tested, the cytotoxicity from ibrutinib was likely independent of its effects on BTK. Supporting this contention, of the tested AML cell lines, KG1a cells were the most sensitive to ibrutinib and yet lacked detectable BTK by immunoblot. Moreover, the IC_50_ in TEX and OCI-AML2 leukemia cells were over 10-fold higher than the concentration of ibrutinib required to completely repress BTK phosphorylation and higher than the pharmacologically achievable concentrations in humans. Consistent with these observations, ibrutinib has been reported to induce AML cell death via a BTK-independent mechanism [8].

Although ibrutinib was largely inactive as a single agent in the tested AML cells, we successfully sensitized two of the more resistant AML cell lines (TEX, IC_50_ = 13.01 μM and OCI-AML2, IC_50_ = 27.44 μM) to ibrutinib by combining the drug with the putative PARG inhibitor ethacridine. However, the observed synergistic cytotoxicity was independent of the inhibitory effect of ibrutinib on BTK. These findings suggest that ibrutinib may also have anti-AML activity that extends to targets beyond BTK.

In addition to inhibiting BTK, ibrutinib cross-reacts with TEC and SRC family kinases with similar efficacy [17]. We noted that the SRC inhibitor dasatinib did not recapitulate ibrutinib's synergy with ethacridine, however the BTK inhibitors ONO-4059 and CC-292, which have reported inhibitory activity against TEC family kinases [23–27], did synergize with ethacridine. We therefore favor the TEC family kinases as likely targets of ibrutinib in its synergy with ethacridine in AML. To date, 5 TEC family members have been identified: BTK, BMX, TEC, ITK, and RLK [28]. ITK is expressed selectively in T cells and its inhibition by ibrutinib leads to decreased STAT6, IkBa, JUNB, and NFAT activity, as well as decreased intracellular calcium release [29]. BMX is expressed in hematopoietic progenitor cells and myeloid leukemias [30, 31] and has been found to mediate STAT3 activation and subsequent transformation by Src [32]. Interestingly, BMX, TEC, and RLK are all expressed in AML and Jurkat D1.1 cell lines (Figure S9). However, further investigations will be required to determine whether these TEC family members—or different kinases altogether—are targets of ibrutinib that explain its synergy with ethacridine. One possible strategy for ibrutinib target determination is a synthetic-lethal human kinome shRNA array, which would uncover kinases that when individually knocked down induce AML cell line sensitivity to ethacridine.

To our knowledge, this work is the first to report on the activity of PARG inhibitors such as ethacridine and gallotannin in AML. While further work is required to fully determine the impact of PARG inhibition in AML, it is interesting to speculate whether some of the single agent activity of ibrutinib in primary AML might be observed in patients with the lowest basal PARG expression.

The addition of poly(ADP-ribose) (PAR) moieties to target proteins alters their structure, function, and localization. PAR-ylation of target proteins is mediated by the PARP (Poly(ADP-ribose) polymerase) family of enzymes, of which PARP-1 is the most abundant and best characterized [33–35]. PARP adds PAR groups to target proteins, and these moieties are removed by PARG [36]. Thus, genetic or chemical inhibition of PARG leads to the accumulation of excess PAR-ylated proteins. Through the accumulation of excess PAR-ylated proteins, PARG inhibition reduces the proliferation of malignant cells [37, 38], sensitizes cells to genotoxic stress [39–41] and inhibits cell signaling pathways including NFκB, p38 and ERK [38]. Through these and other mechanisms, increased levels of PAR-ylated proteins may also promote ROS generation [42], which is relevant to our observed mechanism of action of the drug combination

Though the ibrutinib-PARG inhibitor combination produced striking synergistic cytotoxicity in AML cell lines, it is important to note that this combination also induced cytotoxicity in a subset of normal hematopoietic cells (Figure 3). This observation highlights a potential toxicity that would need to be assessed in the context of clinical trials of these agents.

Thus in summary, through identification of an ibrutinib combination that sensitizes resistant AML cell lines to this kinase inhibitor, we uncovered a novel BTK-independent role for ibrutinib in AML. Moreover, we present a potential role for PARG inhibition as a novel target for combination therapy in AML.

## MATERIALS AND METHODS

### Materials

BTK and GAPDH antibodies were purchased from Cell Signaling Technology (Danvers, MA), BTK pTyr-223 antibody was obtained from Abcam (Cambridge, MA), and β-actin antibody was purchased from Santa Cruz Biotechnology (Santa Cruz, CA). HRP-conjugated goat anti-rabbit and goat anti-mouse secondary antibodies were acquired from GE Healthcare (Buckinghamshire, UK). Kinase inhibitors ibrutinib, CC-292, ONO–4059, PIM1/2, and STO-609 were provided by the Ontario Institute for Cancer Research (Toronto, ON, Canada). Ibrutinib was also obtained from Selleckchem (Houston, TX), as was olaparib. Z-VAD-FMK was purchased from Enzo Life Sciences (Farmingdale, NY). Ethacridine lactate, gallotannin, sulforhodamine-B, hydrogen peroxide, puromycin, and shRNA plasmid-containing bacterial glycerol stocks were purchased from Sigma-Aldrich (St. Louis, MO). The library of internationally prescribed drugs was purchased from MicroSource Discovery Systems, Inc. (Gaylordsville, CT). Alamar Blue was purchased from Life Technologies (Carlsbad, CA) and carboxy-H_2_DCFDA-FITC and MitoSOX Red were obtained from Molecular Probes/Life Technologies (Eugene, OR).

### Cell culture

TEX cells [43] were provided by Dr. John Dick (Ontario Cancer Institute, Toronto, Canada) and grown in Iscove's Modified Dulbecco's Medium (IMDM) supplemented with 15% fetal bovine serum (FBS) (Seradigm/VWR, Radford, PA), 100 μg/ml penicillin, 100 U/ml streptomycin, 2 mM L-glutamine (Life Technologies, Carlsbad, CA), 20 ng/ml SCF (Miltenyi Biotec, San Diego, CA), and 2 ng/ml IL-3 (R&D Systems, Minneapolis, MN). OCI-AML2, NB4, KG1a, Daudi, Thp1, and U937 cells were provided by Dr. Mark Minden (Ontario Cancer Institute, Toronto, Canada). K562 and HL60 cells were provided by Dr. Suzanne Kamel-Reid (Ontario Cancer Institute, Toronto, Canada) and Jurkat D1.1 cells were provided by Dr. Pamela Ohashi (Ontario Cancer Institute, Toronto, Canada). OCI-AML2, K562, Thp1, and NB4 cells were grown in IMDM supplemented with 10% FBS, 100 μg/ml penicillin, and 100 U/ml streptomycin. Daudi, Jurkat D1.1, KG1a, U937, and HL60 cells were grown in RPMI 1640 supplemented with 10% FBS, 100 μg/ml penicillin, and 100 U/ml streptomycin. All cell lines were maintained at 37°C and 5% CO_2_.

### Primary cells

Bulk AML cells from AML patients and peripheral blood stem cells from healthy G-CSF-treated stem cell donors were isolated by Ficoll density centrifugation and apheresis, respectively. Isolated cells were maintained in IMDM supplemented with 10% FBS, 100 μg/ml penicillin, and 100 U/ml streptomycin, at 37°C and 5% CO_2_. All samples were obtained from consenting patients. The collection and use of human tissue for this study were approved by the University Health Network (Toronto, Canada) institutional review board.

### *In vivo* combination treatment

Animal studies were carried out with the approval of the Princess Margaret Cancer Centre ethics review board, and in accordance with Canadian Council on Animal Care regulations. SCID mice were subcutaneously injected with 1 × 10^6^ OCI-AML2 cells. Once tumors were palpable (8 days following injection), mice were treated with ibrutinib (300 mg/kg), ethacridine (20 mg/kg), both in combination (300 mg/kg ibrutinib + 20 mg/kg ethacridine), alongside vehicle control once per day, 5 days/week, for a total of 9 treatments. Mice were subsequently sacrificed and tumor volumes were measured.

### Immunoblotting

Cells were washed twice with 1xPBS and lysed in 1xLaemmli or radioimmunoprecipitation assay (RIPA) buffer. Following quantification with the DC Assay (1xLaemmli) or the Bradford assay (RIPA), protein lysates were resolved by SDS-PAGE and transferred to a PVDF membrane. Membranes were blocked for 1 hour at room temperature in 5% milk-TBST. Blotting with primary antibody was carried out in 5% milk-TBST or 5% BSA-TBST overnight at 4°C. Membranes were then incubated with HRP-conjugated secondary antibody (GE Healthcare, Buckinghamshire, UK) for one hour at room temperature. Proteins were detected by HRP chemiluminescence.

### Cell growth and viability assays

Cells were treated with drug(s) for 72 hours in a 96-well flat-bottomed, clear microplate. Cell growth and viability was determined by the Alamar Blue assay as per the manufacturers instructions or the sulforhodamine-B (SRB) assay as previously described [44]. Cell viability and apoptosis was measured by staining cells with Annexin V-FITC (BioVision, Milpitas, CA) and propidium iodide-PE (Sigma-Aldrich, St. Louis, MO) as per manufacturer's instructions. All flow cytometry experiments were carried out using Canto II 96w or Fortessa LSR X20 cytometers (BD Biosciences, San Jose, CA). Flow cytometry data were analyzed with FlowJo version 7.6.5 (TreeStar, Ashland, OR).

### Combination high-throughput screen

TEX and OCI-AML2 cells were treated with ibrutinib at its respective IC_10_ and IC_25_ values, alongside vehicle (DMSO) controls. Ibrutinib- and vehicle-treated cells were also treated with a library of known drugs at concentrations of 0.133 μM, 1.6 μM, 3.3 μM, 6.7 μM, and/or 13.3 μM. Combination-treated TEX and OCI-AML2 cells were incubated for 72 h at 37°C and 5% CO_2_. The read-out for this assay was percent growth and viability, measured with the sulforhodamine-B (SRB) assay. These data were then used to calculate synergy according to Excess-over-Bliss additivism criteria.

### Excess-over-Bliss additivism for calculating synergy

Excess-over-Bliss additivism (EOBA) [45] provides an estimate of resultant cytotoxicity when two drugs are combined. According to this model, any cytotoxicity unaccounted for by the added effects of both drugs is due to synergy between the two compounds. The formula for excess-over-Bliss is as follows:

EOBA = C − (A + B − (A × B))

where C is equal to the fractional inhibition of both drugs simultaneously, A is equal to the fractional inhibition of drug A, and B is equal to the fractional inhibition of drug B. Fractional inhibition is equal to 1.0 minus the viability (expressed as a value from 0.0–1.0). Positive EOBA values reflect a synergistic combination; the more positive the difference, the greater the synergy. Negative EOBA values reflect an antagonistic drug combination, while near-zero EOBA scores are indicative of an additive drug combination.

### Intracellular and mitochondrial reactive oxygen species measurement

Intracellular reactive oxygen species (ROS) in TEX and OCI-AML2 cells was measured by carboxy-H_2_DCFDA staining on flow cytometry. Cells were stained with 10 μM carboxy-H_2_DCFDA (dissolved in 100% ethanol) and incubated for 30 minutes at 37°C and 5% CO_2_. Dead cells were excluded by propidium iodide (PI) staining. Fold change in intracellular reactive oxygen species production was calculated by dividing the geometric mean of H_2_DCFDA^+^, PI^−^-staining treated cells by the geometric mean of H_2_DCFDA^+^, PI^−^-staining untreated (vehicle-treated) cells. Mitochondrial ROS was evaluated by the same procedure, using 5 μM MitoSOX (dissolved in DMSO) and Annexin V staining for dead cell discrimination.

### shRNA knockdown experiments

Stable knockdown of BTK in TEX and OCI-AML2 was achieved using lentiviral transduction of short hairpin RNAs (shRNA) delivered by the PLKO.1 vector, which contains a puromycin resistance gene. A 72-hour puromycin selection (2 μg/mL in TEX, and 1 μg/mL in OCI-AML2) was used to select for transduced cells 24 hours after lentiviral infection. Following completion of puromycin selection, knockdown was confirmed by immunoblot. The following shRNA sequences directed against BTK (Accession No. NM_000061) were used: shRNA-BTK_974: 5′-GAAGCAGAAGACTCCATAGAACTCGAGTTCTATGGAGTCTTCTGCTTC-3′, and shRNA-BTK_1066: 5′-AGGAGGTTTCATTGTCAGAGACTCGAGTCTCTGACAATGAAACCTCCT-3′.

### PARG activity assay

The HT Universal Colorimetric PARG Assay Kit (Trevigen, Gaithersburg, MD) was used to measure the PARG inhibitor activity of ethacridine. The assay was carried out as per manufacturer instructions.

### Statistical analysis

All graphed viability data are expressed as mean ± SD. Statistical significance was determined by the unpaired Student's *t* test with Holm-Sidak correction for multiple comparisons or a one-way ANOVA with Tukey's post-hoc test for multiple comparisons. Statistical tests were performed using GraphPad Prism 6.03 software (La Jolla, CA).

## SUPPLEMENTARY MATERIALS TABLE AND FIGURES


