# Supplementary material for: Ibrutinib synergizes with poly(ADP-ribose) glycohydrolase inhibitors to induce cell death in AML cells via a BTK-independent mechanism
Source: Oncotarget. 2015 Nov 27;7(3):2765–79. doi: 10.18632/oncotarget.6409 (PMC4823070; doi:10.18632/oncotarget.6409)
Supplement: Supplementary file 1 [file oncotarget-07-2765-s001.pdf]

Ibrutinib synergizes with poly(ADP-ribose) glycohydrolase inhibitors to induce cell death in AML cells via a BTK-independent mechanism

Supplementary Materials

Supplementary Table S1: AML patient characteristics

Patient Characteristics					
Sample ID Diagnosis	Diagnosis	Age at Diagnosis	Gender	Cytogenetics	Molecular
130794	AML	73	Male	46, XY [20]	Not done
130819	AML, M5a	63	Female	46, XX [20]	NPM1+, FLT3–ITD−, FLT3–TKD–
130826	AML, M4	58	Male	46, XY [20]	NPM1+, FLT3–ITD +, FLT3–TKD–
130874	AML	69	Male	49, XY, + 12, + 16, + 21 [10]	Not done
130877	AML	75	Male	48, XY, + 9, + 13 [4]/46, XY [16]	Not done
140994	AML	67	Male	45, XY, −7 [10]	NPM1–, FLT3–ITD–, FLT3–TKD–
141130	AML, M5b	80	Female	Inconclusive	CBFB–MYH11–
150177	AML	53	Female	42~46, XX, –2, der (3) add (3) (p21)? del (3) (q21q26), del (5) (q12), der (7) t (7;?11) (p13;q13), del (8) (p21), add (11) (q13), –18, add (20) (p13), +3 mar [4]	Not done
150256	AML	24	Male	45, XY, der (6;7) t (6;7) (p21;q22) del (6) (q13q21) [17]/46, XY [3]	Not done

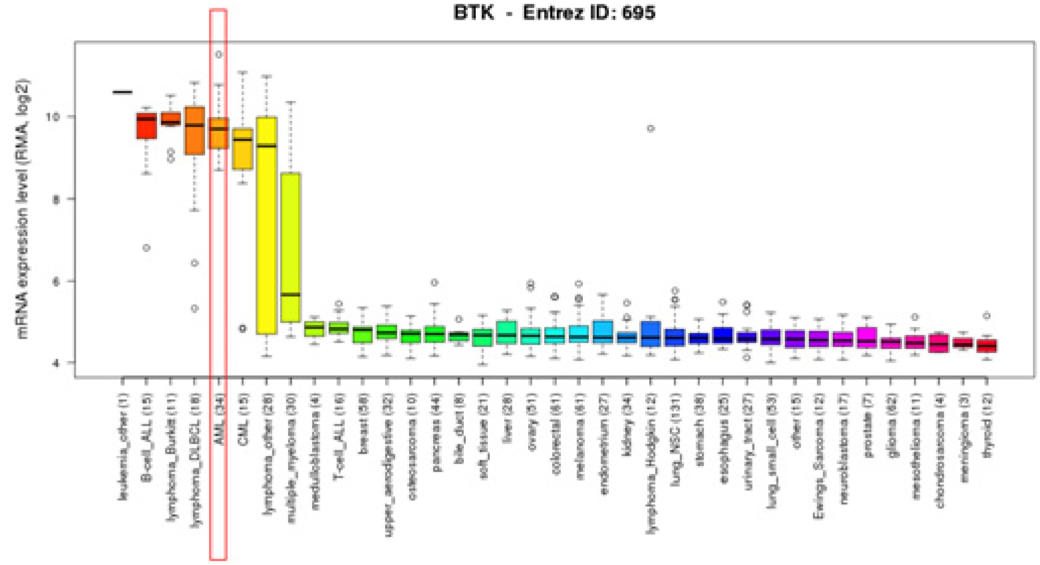

Supplementary Figure S1: BTK mRNA levels in AML cell lines are similar to those of B-cell malignancies. BTK mRNA expression in cancer cell lines, based on the Cancer Cell Line Encyclopedia [4].

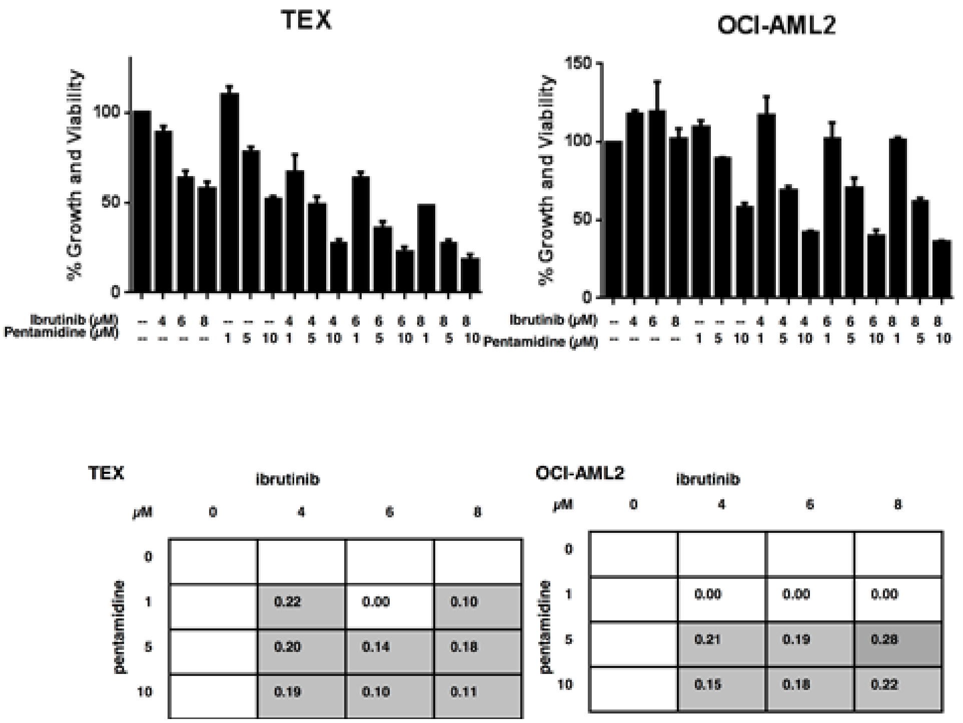

Supplementary Figure S2: Combination chemical screen validation for pentamidine. TEX and OCI-AML2 cells were subjected to 72 h treatment with concentrations of ibrutinib and pentamidine similar to those tested during the combination chemical screen. Cell growth and viability was measured with the SRB assay, and calculated relative to untreated cells. Data represent mean percent growth and viability ± SD and mean EOBA scores from a single experiment performed in triplicate.

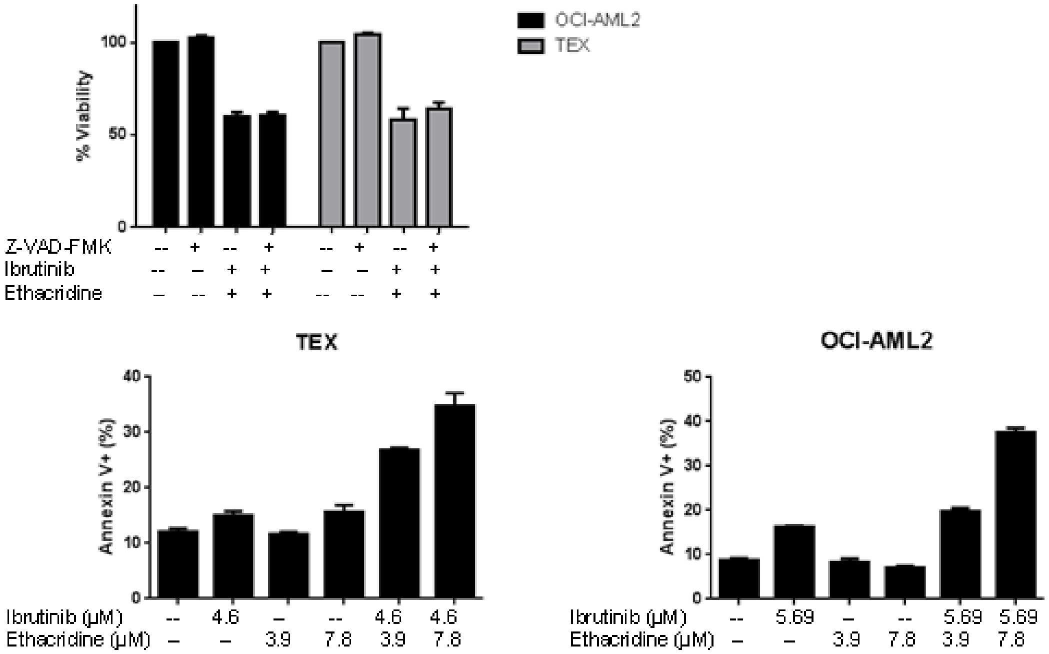

Supplementary Figure S3: Cell death caused by ibrutinib-ethacridine combination is caspase independent. Top: TEX and OCI-AML2 cells were subjected to combination ibrutinib (4 µM)-ethacridine (6 µM) treatment in the presence and absence of 50 µM Z-VAD-FMK (caspase inhibitor) for 48 h. Viability was subsequently measured with Annexin V and PI staining on flow cytometry and calculated relative to vehicle-treated cells. Bottom: TEX and OCI-AML2 cells were treated at the indicated concentrations of ibrutinib and/or ethacridine for 48h, and induction of apoptosis was measured by Annexin V staining on flow cytometry.

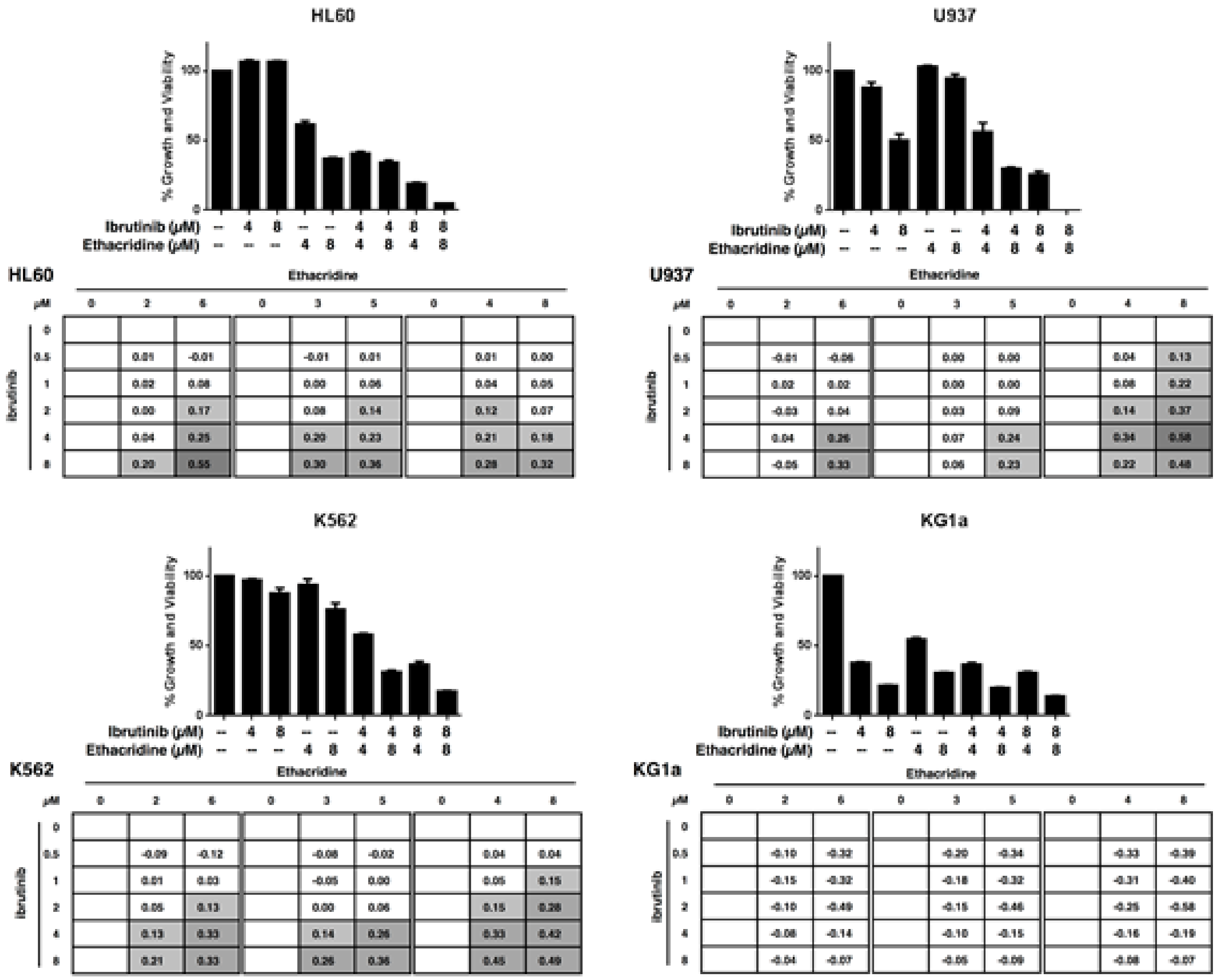

Supplementary Figure S4: The ibrutinib-ethacridine combination is strongly synergistic in HL60, U937, and K562, but not KG1a AML cell lines. AML cell lines were treated with increasing concentrations of ibrutinib and ethacridine for 72 h. Relative growth and viability was measured with the Alamar Blue assay. Data depict mean growth and viability ± SD and mean EOBA scores from a representative experiment performed in triplicate.

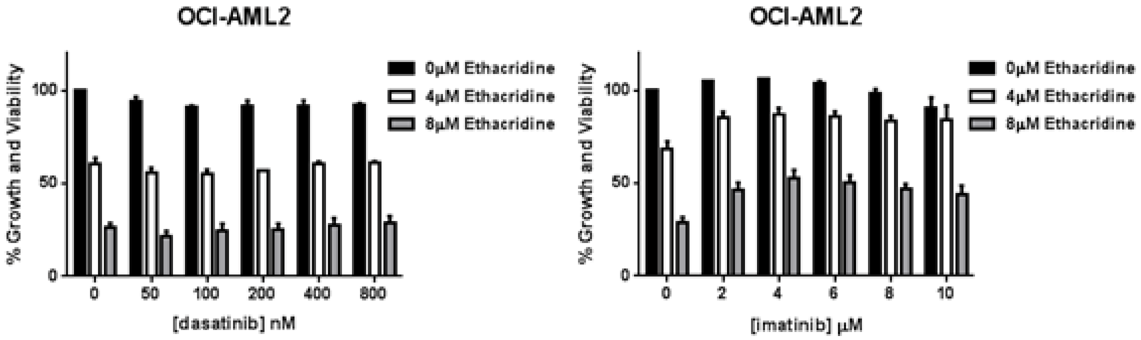

OCI-AML2 Dasatinib (nM)	Ethacridine (μM) 0	4	8
0			
50		0.02	0.03
100		0.00	-0.01
200		-0.01	0.00
400		-0.04	-0.03
800		-0.05	-0.04

OCI-AML2 Imatinib (μM)	Ethacridine (μM) 0	4	8
0			
2		-0.16	-0.18
4		-0.18	-0.24
6		-0.17	-0.22
8		-0.16	-0.19
10		-0.22	-0.18

Supplementary Figure S5: Dasatinib and imatinib do not synergize with ethacridine in OCI-AML2 cells. OCI-AML2 cells were combination-treated with ethacridine and dasatinib (left) or imatinib (right) for 72 h. Cell growth and viability was measured with the Alamar Blue assay, and calculated relative to untreated cells. Synergy was calculated with the EOBA formula. Data represent mean percent growth and viability ± SD and mean EOBA scores from a single experiment performed in triplicate. Data are representative of three independent experiments.

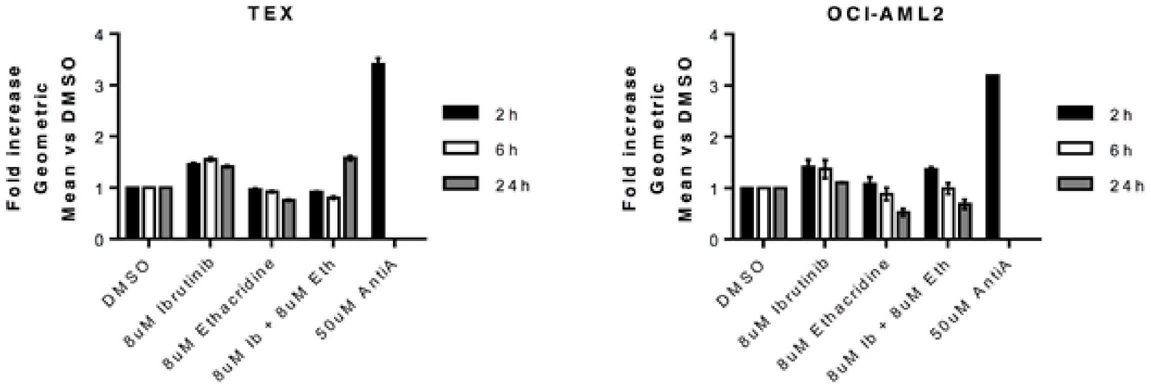

Supplementary Figure S6: Ibrutinib and ethacridine treatment does not produce mitochondrial ROS. TEX and OCI-AML2 cells were treated with ibrutinib, ethacridine, or both drugs in combination for 2, 6 or 24 h. Mitochondrial ROS was measured by MitoSOX Red staining, with dead cell exclusion by Annexin V staining on flow cytometry. Fold increase in mitochondrial ROS was calculated relative to the geometric means of carboxy-H_2DCFDA (FITC) staining in untreated cells. Antimycin A (50 μM) treatment served as a positive control for mitochondrial ROS generation.

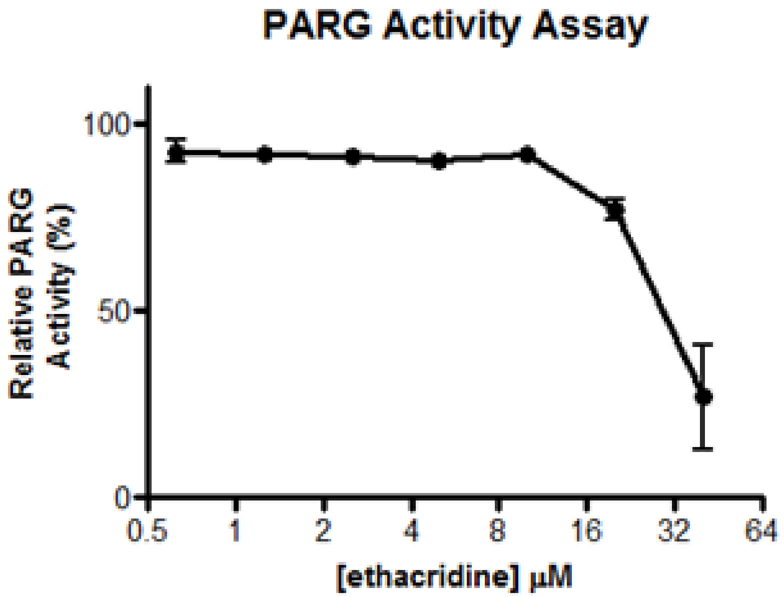

Supplementary Figure S7: Ethacridine is a PARG inhibitor. Ethacridine's inhibitory activity against PARG was determined using a cell-free colorimetric assay that measures levels of biotinylated PAR attached to histones in the presence of PARG enzyme. A loss of absorbance at 450 nm correlates with increased PARG activity. Relative PARG activity was calculated by comparing the loss of absorbance at 450nm in the presence of ethacridine to that of no PARG control (maximal absorbance at 450 nm). Data represent mean PARG activity ± SD from a single experiment performed in triplicate.

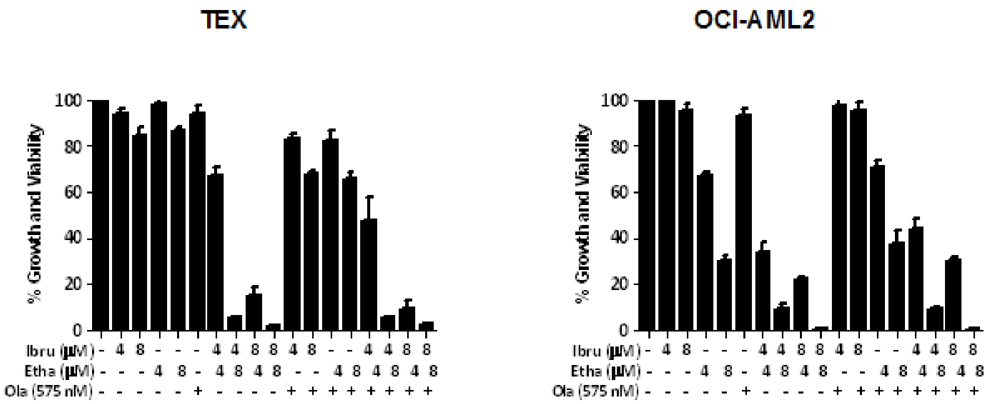

Supplementary Figure S8: Treatment of TEX and OCI-AML2 cells with olaparib in combination with ibrutinib and ethacridine. TEX and OCI-AML2 cells were pre-treated with the PARP inhibitor olaparib 4 hours prior to a 72 h incubation with ibrutinib, ethacridine or both in combination at the indicated concentrations. Growth and viability was measured by the Alamar Blue assay and then calculated relative to untreated controls.

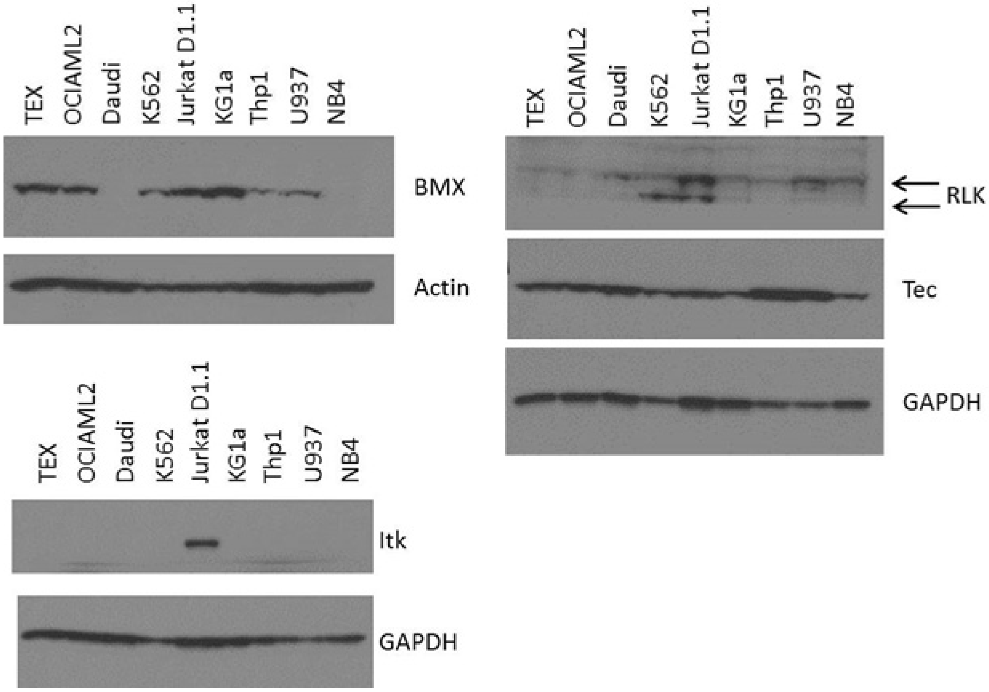

Supplementary Figure S9: Expression of TEC family kinases in AML cell lines. Expression of kinases BMX, TLK, TEC, and ITK in a panel of AML cell lines, Jurkat D1.1 and Daudi cells was determined by immunoblotting.
